# Dhaka landfill waste practices: addressing urban pollution and health hazards

**DOI:** 10.5334/bc.108

**Published:** 2021-07-28

**Authors:** Salma Akter Urme, Marzuka Ahmad Radia, Rafiul Alam, Mohammed Uzzal Chowdhury, Shahriar Hasan, Shakil Ahmed, Hasna Hena Sara, Mohammed Syful Islam, Delufa Tuz Jerin, Prianka Sultana Hema, Monybur Rahman, A.K.M. Mazharul Islam, Mohammed Tanvir Hasan, Zahidul Quayyum

**Affiliations:** James P. Grant School of Public Health, BRAC University, Dhaka, Bangladesh; James P. Grant School of Public Health, BRAC University, Dhaka, Bangladesh; Disabled Rehabilitation and Research Association (DRRA), Dhaka, Bangladesh; James P. Grant School of Public Health, BRAC University, Dhaka, Bangladesh; James P. Grant School of Public Health, BRAC University, Dhaka, Bangladesh; Jatiya Kabi Kazi Nazrul Islam University, Trishal, Mymensingh, Bangladesh; James P. Grant School of Public Health, BRAC University, Dhaka, Bangladesh; James P. Grant School of Public Health, BRAC University, Dhaka, Bangladesh; Department of Anthropology, Shahjalal University of Science and Technology (SUST), Sylhet, Bangladesh; James P. Grant School of Public Health, BRAC University, Dhaka, Bangladesh

**Keywords:** cities, environmental health, hazard, landfill, pollution, public health, urban, waste management, Dhaka, Bangladesh

## Abstract

Two Dhaka, Bangladesh, landfills are explored to understand how management practices impact environmental quality and public health in the surrounding areas. A combination of research methods is used, such as geospatial buffer zone analysis, semi-structured observation checklist and qualitative interviews, to gain an understanding of the waste transportation, leachate percolation, and adverse health and environmental effects. A multi-ring buffer zone and ground truth method were applied through ArcMap for the spatial distribution of landfill-adjacent environmental features. Qualitative interviews were conducted with landfill officials and nearby residents. Findings reveal that landfills are situated very close to residential areas, water bodies and agricultural lands, exposing them to various health and environmental hazards. Improper solid waste management practices of the landfills cause adverse environmental effects by leachate percolation, waste incineration and vector breeding. Adjacent dwellers suffer from bronchial diseases, pneumonia, diarrhoea, itching problems, headache and appetite loss. The existing solid waste management system requires managerial and technical modifications to reduce the associated environmental pollution and health hazards.

## 1 Background

Rapid urbanisation and industrialisation, the main elements of worldwide economic and social development, are also interlinked with rising waste generation (Vij 2012). The generated waste must be managed to ensure resource conservation and protection of human health and the environment (Pongrácz & Pohjola 1999). Waste management can be defined as the collection, transport, recovery and disposal of waste, including the supervision of such operations and aftercare of disposal sites such as landfills (European Council 1991).

Landfills are currently the most common method of waste disposal (Kaza *et al*. 2018). A landfill is defined as a large area of land or excavated site specifically designed and built as the final disposal site of solid municipal waste (Abdel-Shafy & Mansour 2018). Globally, approximately 37% of waste is disposed in landfills (Kaza *et al*. 2018), with approximately 52.6% of waste landfilled in the US, 59.1% in Brazil, 94.5% in Malaysia, 79% in China (Vaverkova 2019) and 42% in Bangladesh (Amin 2017).

Research has shown that the waste management process in landfills is associated with surface and groundwater contamination by landfill leachate, pungent odours, bio-aerosol emissions and hazardous organic compounds (Environment Victoria 2013; Garrod & Willis 1998; Sakawi *et al*. 2011, De Feo *et al*. 2013; Maqbool *et al*. 2011; Butt & Ghaffar 2012). Landfill leachate percolates into groundwater or surface water through flaws in the liners, posing a major problem for aquifers (El-Salam & Ismail 2013).

Many developing Asian cities, including Dhaka, Bangladesh, face severe problems in managing increasing levels of solid waste generated by the growing urban population (Idris *et al*. 2004). Currently. Dhaka’s limited waste management system is dealing with a high volume of waste generation, approximately 4500 tonnes/day (Mahmud 2018). Currently, two landfills in Matuail and Aminbazar serve the Dhaka South City Corporation (DSCC) and Dhaka North City Corporation (DNCC), respectively. Before 2006, only the Matuail landfill (under the DSCC) had been established by the Japan International Cooperation Agency (JICA) and Japan Debt Cancellation Fund (JDCF). In 2006, with funding assistance from the JICA, another landfill was built at Aminbazar (under the DNCC), and it is still operational, despite exhausting its capacity in 2017. This funding was extended as aid, and the JICA is not in charge of any of the landfill operations or management activities, although it collaborates with both the DNCC and DSCC regarding waste management activities conducted in the landfill.

The solid waste deposited in landfills may cause adverse effects on the surrounding environment and people living close to landfill sites (Njoku *et al*. 2019). The effect of composting on land and landfill gas generation, landfill location, treatment of leachate, and leachate pollution are pertinent issues addressed in previous studies (Hai & Ali 2005; Azim *et al*. 2011). Common health effects of living near a landfill include low birth weight, congenital anomalies and respiratory diseases (Shaddick *et al*. 2018). Brender *et al*. (2011) showed significant correlation between residential proximity to environmental hazards and adverse health outcomes, especially risks for central nervous system defects, congenital heart defects, low birth weight, cancer, asthma, chronic respiratory symptoms, *etc. A* study on South Africa also mentioned that living within 5 km of a waste site was significantly associated with increased risks of tuberculosis, asthma, diabetes and depression (Tomita *et al*. 2020). However, there is a lack of studies focusing on the health risk of the population directly involved with waste management and the environmental impacts of Dhaka’s landfills.

To address the gaps in existing studies, the aims of this paper are as follows:

- To identify the spatial locations of Dhaka’s landfill sites and their surrounding areas that may be exposed to environmental pollution.
- To identify the limitations of the current landfill waste management system, including the maintenance and recirculation process of leachate.
- To explore the effect of solid waste disposal on the health of nearby residents.

The Pathways to Equitable Healthy Cities project conducted a study between July 2019 and January 2020 on the topic of municipal solid waste management (SWM). This study adopted a combination of research methods (literature review, primary and secondary data collection and analysis) to obtain a deeper understanding of the different issues surrounding Dhaka’s municipal waste. The study explored the methods of primary SWM (generation, collection, segregation and disposal) used by the city dwellers, waste transportation and management at secondary transfer stations (STSs) and the final landfill waste disposal process. The study also explored the effects of improper practices on urban health and environment.

This paper focuses on a sub-study of the above-mentioned research, focusing on waste management of the two landfills at Aminbazar and Matuail managed by DNCC and DSCC, respectively.

## 2 Materials and Methods

This paper uses qualitative research methods such as key informant interviews (KIIs), focus group discussions (FGDs) and qualitative observation checklists. KII respondents included landfill officials with more than six months of job experience, and SWM professionals, researchers, academicians and civil society experts engaged with SWM activities. Ten KIIs and two FGDs were conducted in total. For the FGDs, respondents were included who had experience of living beside the landfill for at least six months prior to the interview. For each FGD, a balanced group of eight to 10 respondents ensuring gender, age and profession sensitivity were selected. This study used a purposive sampling method for the identification of study respondents and conducted interviews after obtaining their consent. An official from the Matuail landfill was interviewed as a KII respondent, however lack of permanent managerial staff at Aminbazar prevented the authors from conducting a KII at Aminbazar. The collected data (preserved in field notes, jottings and recordings) were precisely reviewed and analysed to identify the underlying meaning of the respondent quotations. Thematic analysis was conducted using NVivo by assigning codes to the emerging key themes and patterns through systematic examination.

An observation checklist was used to assess the overall landfill scenario. The key issues for the assessment included: (1) the overall waste management framework; (2) the leachate collection and treatment plant; (3) the method for measuring the dumped waste; (4) the dumping platform; (5) the waste segregation practice; (6) swarming mosquitoes, flies, hawks and crows; (7) waste incineration; and (8) protection of the landfill area and exposure of the surrounding areas to landfill hazards.

A multi-ring buffer zone and ground truth method for feature identification were applied through ArcMap software to represent the spatial distribution of the adjacent environmental features of the two landfills.

## 3 Findings

This section thematically presents the findings of the study. The identified themes—including loss of land and land ownership issues, landfill location, the management and waste treatment process, including the leachate percolation chain, environmental and soil contamination of the landfill’s surrounding areas, and the health risks of landfill workers and neighbouring dwellers—are elaborated on below.

### 3.1 Location of the Landfills

The Aminbazar landfill under the DNCC, situated in Savar upazila, 24 km north-west of Dhaka city, became operational in 2007 over 52 acres (21 ha) of land. This landfill covers almost five zones and 36 wards of the DNCC. The other landfill at Matuail, administered by the DSCC, about 8 km from Gulistan under the Matuail Union to the south of Dhaka, was established as an open dumping station of 50 acres (20 ha) in 1995, and 50 additional acres (20 ha) were added in 2006. It covers 57 wards under five zones of the DSCC.

**Figure 1:**
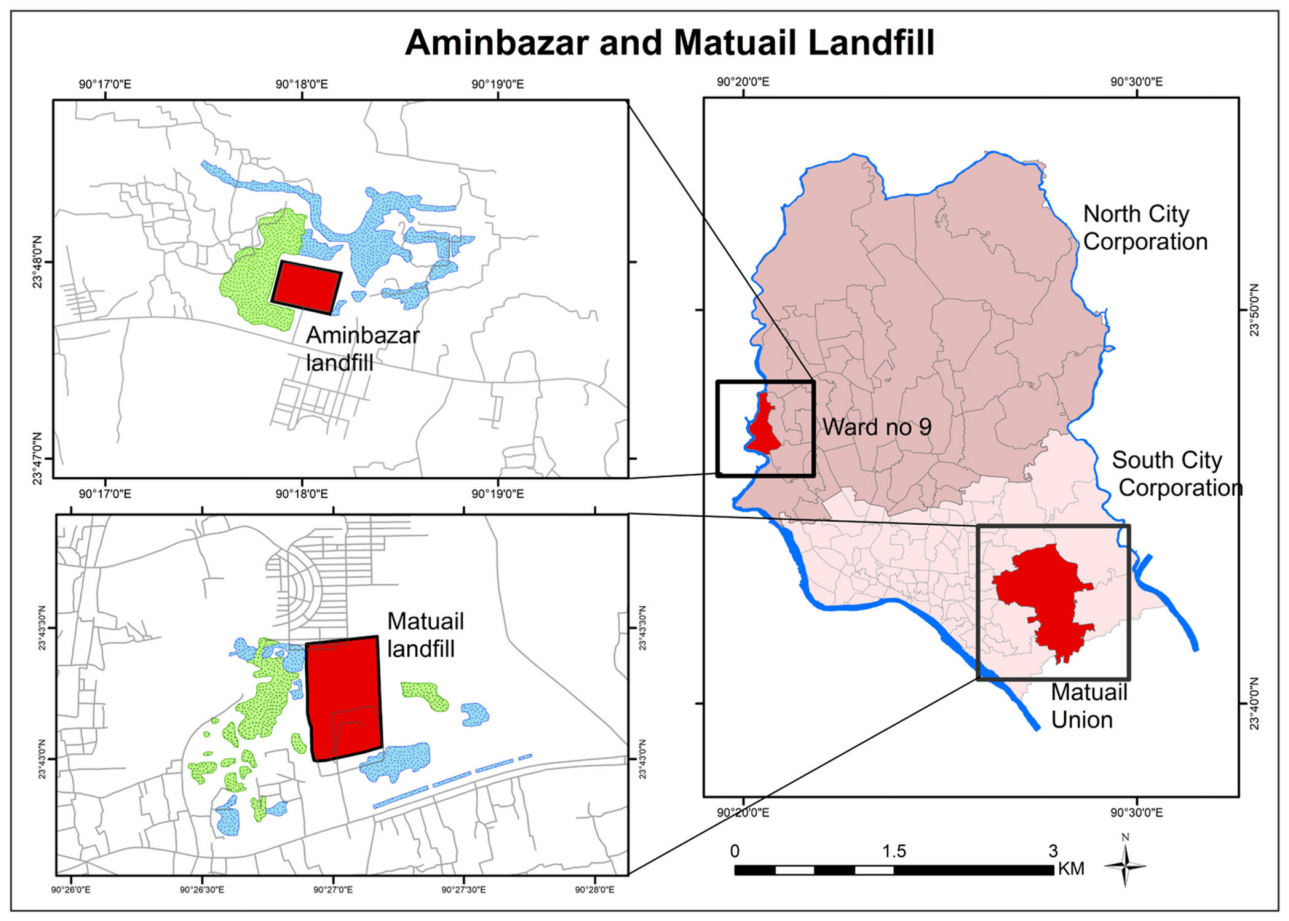
Locations of the study areas of the Aminbazar and Matuail landfills.

The tertiary waste treatment of Dhaka city occurs in the Matuail and Aminbazar landfills. The Matuail landfill is approximately 300 m from the central highway of Matuail, in the south-eastern part of Dhaka and approximately 3.75 km from the centre point Gulistan of Dhaka. This landfill occupies almost 100 acres (40.5 ha), with the authorities trying to acquire an extra 81 acres (32.8 ha). At the other end of Dhaka, the Aminbazar landfill is approximately 1 km from the central highway under ward number 9 situated in the north-western part of Dhaka (Figure 1).

**Figure 2:**
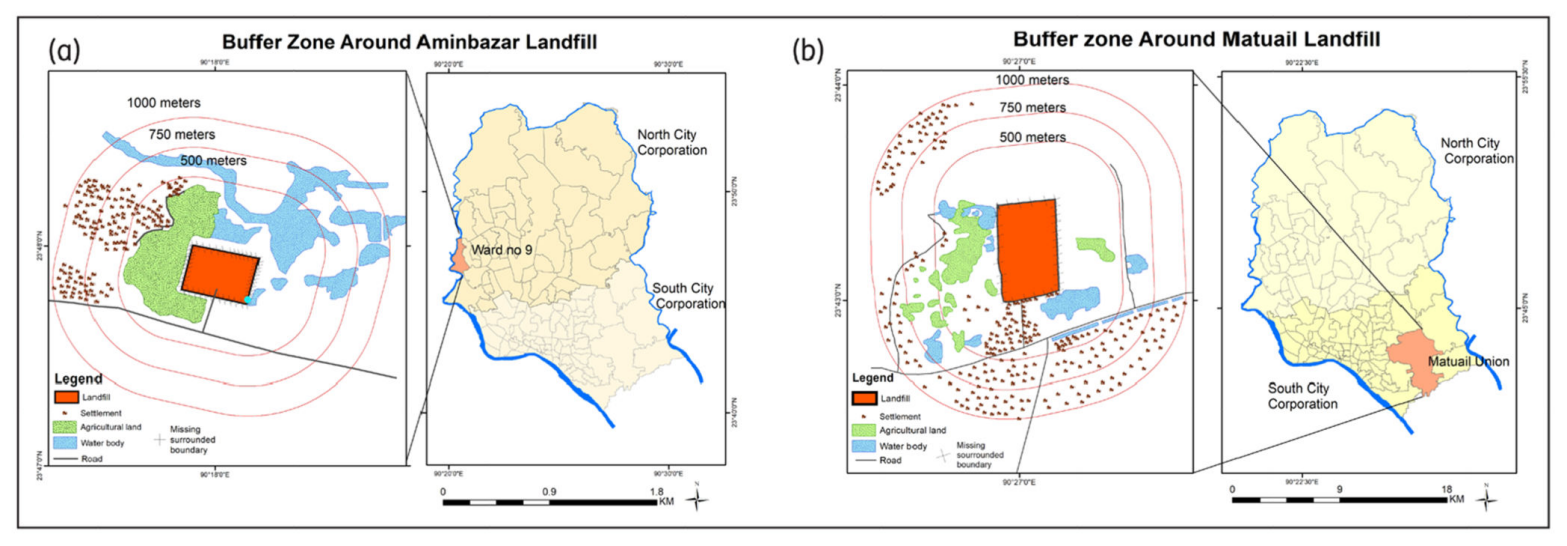
Buffer zones of the (a) Aminbazar and (b) Matuail landfills.

Both landfills are located very close to residential areas, water bodies and agricultural land, approximately 500 m away, exposing these areas to various hazards. Settlement density and built-up areas are situated closer to the Matuail than to the Aminbazar landfill (Figure 2b). However, the Aminbazar landfill is situated on top of a lower flood flow zone with many waterbodies, agricultural fields and a river located nearby. A multi-ring buffer zone analysis using 500, 700 and 1000 m benchmarks (the greater distance represents lesser exposure to pollution) was used to show the assigned distance from the two landfills. This analysis shows the spatial distribution to environmental features, *e.g*. water bodies, agricultural land, the river and other built-up areas, and the likelihood of landfill-induced exposure. Figure 2 represents this analysis for Aminbazar and Matuail, respectively.

Figure 2a illustrates a large area of agricultural land within the 500 m benchmark, a highly vulnerable zone. This agricultural land, susceptible to leachate permeation, supports the livelihood of many neighbouring dwellers. Additionally, many shallow water bodies within the 500 m buffer zone on the eastern side and many settlements within the 500–1000 m benchmark can be observed.

**Figure 3:**
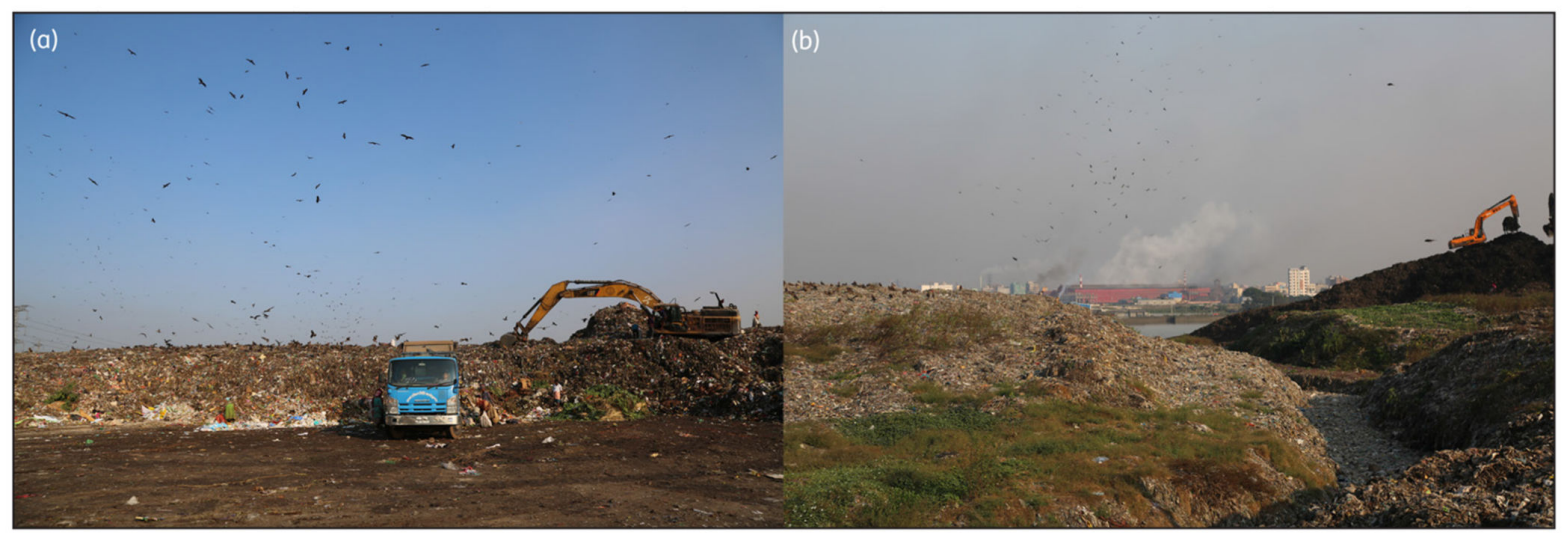
Peripheral views of the (a) Aminbazar (dumping platform) and (b) Matuail landfills.

Figure 2b depicts the distance of the mentioned topographies from the Matuail landfill. Although the adjacent areas of the landfill do not have as many waterbodies and agricultural land as Aminbazar, many settlements and waterbodies are located within the 500 m benchmark, especially on the south and south-western sides. A peripheral view of the Aminbazar and Matuail landfills is shown in Figure 3.

### 3.2 Landfill Management

Aminbazar has around 25 permanent workers to ensure the smooth operation of the whole landfill, and management is overseen through sporadic visits by city corporation officials. No appointed landfill-in-charge officer is available. This reflects certain managerial gaps compared with the Matuail landfill, which has 26 permanent workers, including three conservancy inspectors to manage the landfill working in eight-hour shifts. The KII at Matuail revealed that approximately 2000 tonnes of waste are disposed of daily, and in 2018 and 2017, almost 947,000 and 800,000 tonnes of waste were dumped in Matuail, respectively.

### 3.3 Land Scarcity

The total area of the Matuail landfill is 99 acres (40 ha), whereas that of Aminbazar is 50 acres (20.2 ha). The Waste Database 2014 stated that 388.5 acres (157.2 ha) of land are required to accommodate 8,646,120 tonnes (waste generation in 2014) of waste per year, or 23,688 tonnes/day for the whole city (Waste Concern 2014). Additional land is urgently required to properly manage the rising waste volume. In this regard, a key stakeholder explained that:

Land availability and land ownership are crucial challenges for proper waste management at landfills. For disposing around 1 million tons of waste, Matuail landfill needs 10 acres [4 ha] of land—every day the city generates around 3,000 tons of waste. The waste management situation is exacerbated due to land scarcity.

Both the DNCC and DSCC waste management departments have a plan to acquire land for the expansion of both landfills due to exceeding their existing capacities. A total of 81 and 80 acres (32.8 and 32.4 ha) of land acquisition for Matuail and Aminbazar, respectively, has commenced (Khan 2018; and KII respondents) and is expected to be completed soon.

### 3.4 Loss of Land

Some FGD respondents stated that discarded waste tends to spread outside the designated dumping area due to the absence of a landfill boundary wall. Furthermore, the Aminbazar landfill is established on private land, originally owned by the nearby village people, previously used to cultivate ‘boro’ rice. Some of the landowners are yet to be compensated by the government for their land. They are also exposed to the negative externalities (pungent odour, leachate percolation, discarded waste) generated by landfill operations. While the Aminbazar landfill is managed and monitored by the JICA in collaboration with the city corporation, the DNCC remains in charge of the landfill, including overseeing all financial and resource issues.

During the FGD, the respondents mentioned that the Aminbazar landfill was built in a low flooding zone, considered illegal according to land restrictions. The Bangladesh Environmental Lawyers Association (BELA) is currently fighting a case to stop operations at Aminbazar. Notably, it also complained that the landfill’s authorities were allocated 52 acres (21 ha), but are presently using 73 acres (29.5 ha), with the government trying but failing to maintain the buffer zone.

### 3.5 Waste Disposal in Landfill

According to the study respondents, the municipal solid waste disposed at landfills is collected from the STSs by container carriers, compactors, open trucks or dump trucks, with compactors commonly used in the DNCC and container carriers and open trucks more common in the DSCC. Every day at least 500–550 trucks transfer waste to each landfill. A compactor truck driver stated the need for one to four visits to the Aminbazar landfill per day, based on waste quantity. Approximately 75 minutes are needed for each waste-filled truck to reach the landfill from the STSs, and approximately 24 hours for the waste to reach the landfill from households.

The observation checklist findings reveal that the waste-carrying trucks from the STSs are weighed by the weighing bridge at the entrance of the landfills both before and after dumping the waste to ascertain the exact weight of dumped waste. The registration number is recorded manually. This information is recorded and shared by the DSCC and DNCC. The waste in the dumping platform (an area where the waste is dumped and spread onwards and upwards within the landfill area) is not segregated before dumping. There are five and four dumping platforms at Aminbazar and Matuail, respectively. The dumped waste is rearranged and flattened by excavators, bulldozers, pay loaders and dumpers with tyre dozers, chain dozers and scrapers used for equal distribution.

### 3.6 Waste Segregation in Landfill

The findings revealed that recyclable waste is manually segregated by informal waste pickers in both landfills and sold for their livelihoods. These waste pickers, which are comprised of children, women and men, are not formally employed by the city corporations or any private organisations.

During the observation, it was found that the waste pickers or *‘tokais’* (informal waste collectors, usually children) do not use any protective equipment while separating the recyclable items: plastic bottles or other plastic products, paper, iron utensils, *etc*. Only a few use gumboots. It was also observed that they eat their lunch within the landfill boundary areas, which poses serious health hazards. One stakeholder responded about why protective equipment is not provided to the waste pickers:

We alert them about the importance of using the safety gear. But we can’t provide safety gear to the landfill *‘tokais’* due to budget constraints.

An informal syndication to process recyclable waste materials near the Matuail landfill was also observed. This is run by middlemen paying very low daily wages to a few waste handlers to segregate and gather recyclable products, later sold to the plastic product manufacturers or wholesale vendors by the middlemen. However, the Matuail KII respondent conveyed that they only allow the women and children to collect recyclable items to sustain their livelihood through the following statement:

This is allowed because of humanitarian reasons. They come here to earn something, their family is dependent on them, so this is different […] some of them are collecting the broken glass, some are collecting the plastic […] so they can earn some money and be benefited.

### 3.7 Water Contamination and Leachate Treatment Plant

The methodology of this study impeded carrying out scientific or laboratory tests of groundwater. Through the observation checklists it was found that leachate treatment plants have been introduced at both Aminbazar and Matuail to minimise leachate percolation into ground water. However, qualitative findings revealed that groundwater is still being polluted with leachate.

**Figure 4:**
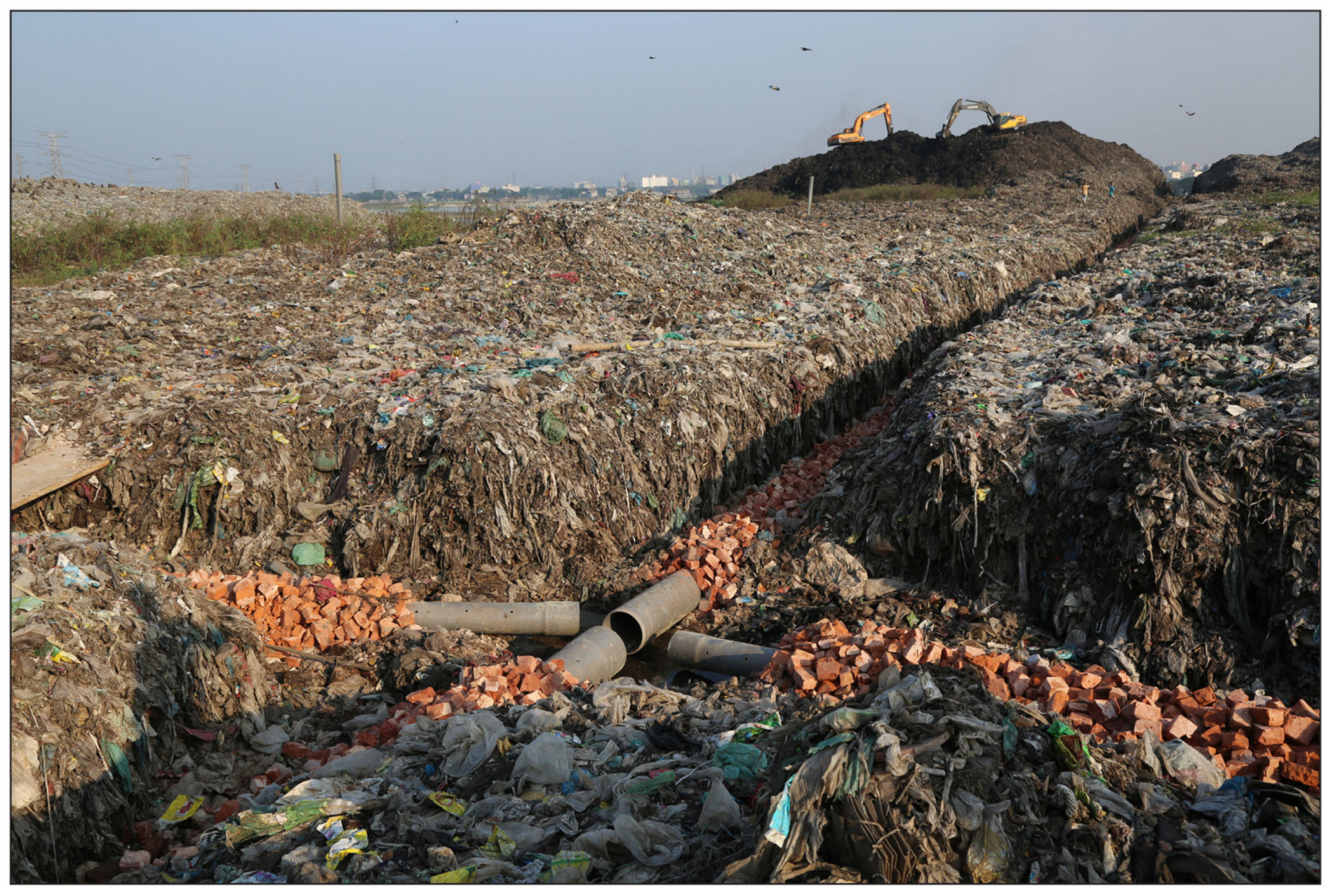
Polyethylene pipes for leachate collection at the Matuail landfill.

The leachate treatment plant in Matuail was established in 2006. The raw leachate moves steadily into the leachate pond through high-density polyethylene pipes configured in a fish skeleton pattern in the dumping platform (Figure 4).

From the qualitative observation checklist, it was found that Matuail’s leachate water treatment plant is divided into two parts, where the raw leachate is accumulated in the raw leachate pond. After filtration, it flows to the semi-aerobic treatment pond. The depth of these two ponds is 15 m. The raw leachate is treated by chemical oxygen demand and biological oxygen demand, with three tanks filled with three types of chemicals: ferrous sulphate (FeSO_4_), lime (CaO) and polymer. In the semi-aerobic pond, bacteria and fish are cultured to treat the water, and a blower machine continuously blows air into the pond. The clean leachate from Matuail is then carried out to the nearby land for crop cultivation after testing for water toxicity through the fish test technique. Fish cultured in the semi-aerobic pond are used to test the water quality before allowing the fresh leachate water to join the mainstream water. This treatment plant works four hours daily, and eight hours daily during the rainy season to counterbalance the additional rainwater mixing with the leachate.

The Aminbazar landfill also has a leachate treatment plant, with a more systematic collection system than Matuail. Originally established in 2012, it became operational from April 2018 with improved, automated and scientific technology. This leachate treatment plant helps to purify the leachate and convert it into safe water. The raw leachate is extracted from the waste through pipes and transferred to the leachate treatment plant by a canal. The raw leachate, accumulated in the raw leachate pond, is broken down by hydro-smart technology into smaller particles, and chemically treated together by FeSO_4_ and CaO. After the breakdown, the coagulation and flocculation processes take place in a separate tank. After the primary purification, the water is further purified in an aerobic pond. In the aerobic treatment plant, bacteria are cultured to treat the water and a blower machine continuously blows air. After cleansing, the treated water is subjected to dissolved oxygen test and can join the mainstream water after satisfactory results are obtained.

It is challenging to properly maintain the leachate pond during the monsoon due to the mixing of rainwater and leachate water. The in-depth FGD findings suggest that the leachate and the waste mixing with the groundwater adversely affects the surrounding water bodies, killing the fish population and hampering fishermen’s livelihoods. One respondent stated:

And […], the river has become ruined by waste entering it. There is [*sic*] no fish here anymore.

Landfill officials assured that the leachate treatment plant is working well. However, the observation from the villages near the Aminbazar landfill as well as the comments of the FGD respondents showed that the leachate treatment plant does not fully reduce leachate percolation into the nearby waterbodies, making them polluted and unsuitable for fish cultivation. Thus, many farmers and fishermen have been forced to switch livelihoods. The existing leachate percolation system needs improvement to reduce adverse environmental and health effects.

### 3.8 Soil Contamination

Crops are cultivated informally in the Matuail landfill by nearby dwellers. While this is an additional income source for the farmers, it is also a probable health risk caused by the heavy metal contents such soils may pose due to the huge volume of dumped waste. An official from the Matuail landfill mentioned:

Just after the winter season, the surrounding agricultural land will depict a completely different scenario, because the farmers will start cultivating the crops at that time.

On the other hand, dwellers beside the Aminbazar landfill cannot cultivate crops due to the soil and groundwater contamination caused by leachate permeation and waste dumping. An FGD respondent stated:

We previously cultivated crops in this land [around the landfill]. But nowadays polythene has been buried in that farming land [a field besides the landfill]. Now all the fields have become barren and we can’t cultivate any crops.

This study also observed that many agricultural lands are located around 200–300 m radius from the dumping zone of the Aminbazar landfill (observable in Figure 2a), which is a highly risky zone. Furthermore, especially during the monsoon, the waste becomes clogged in the farmland, causing soil infertility.

### 3.9 Pungent Odour and Adverse Effects

The present study’s findings reveal a link between pungent odour spread by the waste and adverse health effects. The emission of a foul stench from the landfills is a huge problem for the landfill workers and adjacent households. The FGD respondents mentioned that their lives have become unbearable due to the landfill’s emitted odour. This also negatively affects those walking or travelling along the highway/roads beside the landfills and contaminates the air. No evidence was found from the observation checklist or KIIs of the landfill authorities that the landfill authorities are taking any steps to address the existing odour issue at the landfills.

### 3.10 Mosquito and Animal Infestations

Study observations reveal that many flies sit and breed on the putrid waste. Mosquito breeding is higher within and around the landfills and contributes to the spread of various vector-borne diseases among nearby dwellers. An FGD participant living near the Aminbazar landfill expressed his opinion as follows:

We [the nearby people of the landfill] can’t even stay properly inside the house due to the disturbance of flies and mosquitoes.

Along with the mosquitoes and flies, there are also rodents, dogs, snakes, egrets, hawks and many migrant birds at the landfill. Landfill management workers mentioned that egrets stay from dawn to 10 a.m. and after that kites come and stay up to 4 p.m. They come to feed and consequently destroy the adjacent agricultural crops and spread germs around the landfill areas. A few dwellers near the landfills with sufficient financial resources relocated from the area due to the foul odour. However, most current residents near the landfill are local landowners, making relocation challenging. While the landfill authorities and city corporation officials acknowledge that mosquito infestation in particular is a major problem, and have increased the annual budget for mosquito control activities incorporating the landfills, they are still unable to make a major headway. No evidence was found in terms of the landfill and city corporation authorities taking steps to address the animal infestation issue.

### 3.11 Incineration of Waste

Landfill officials stated that waste incineration is prohibited. However, FGD respondents mentioned that they could observe and smell incinerated waste and smoke from the landfill site. In this regard, a KII respondent from the landfill site mentioned:

No, we don’t burn any waste here, if it ever catches fire, we take immediate action to stop the fire. If the fire isn’t controlled within an hour, it takes about two to three days to bring it under control. It spreads really fast, the waste produces methane gas, so the fire spreads very quickly and very deep, for about 3–4 feet.

But the people living close to the landfill expressed a contrasting view:

Yes, sometimes the waste is burnt, it catches fire, and it seems that the wind goes in that direction […], you can’t survive in the house at that time due to the foul odour coming from the waste.

Confirming the above observation, spontaneous waste incineration in the Aminbazar landfill was observed during the authors’ visit. This incineration emits hazardous smoke which is unhealthy for those living and working around the landfills. The local government has recently taken an initiative to establish a waste-to-electricity plant at Aminbazar, which will require 3000 tons of waste daily, thus reducing the waste scattered across the city. The JICA is currently assessing the feasibility of the project. However, due to lack of waste segregation, ensuring the quality of the energy remains challenging (Prothom Alo 2020). During the landfill visit, it was discovered that the DNCC has initiated a project with the China Machinery Engineering Corporation to set up a 42.5 MW power plant at the landfill to produce electricity by incinerating the waste at a high temperature (The Daily Observer 2020).

### 3.12 Health Risks

People living close to the landfills may suffer from pneumonia, bronchial and skin diseases from the huge amounts of waste dumped there, as reported by the FGD respondents. Nearby dwellers also suffer from regular headaches, stomach problems and a loss of appetite due to the foul smell. Another FGD participant stated:

I cannot eat properly, I lost my appetite after starting to live here [mentioned the landfill] […].

FGD respondents reported suffering from pneumonia, bronchial and skin diseases, with children suffering primarily from pneumonia. Farmers working near the landfill face many injuries, with rashes occurring commonly after submerging their feet in contaminated rivers or agricultural fields. One FGD respondent said:

needles, ceramic chunks or broken glasses pierce the feet of those working near the landfills. They can’t dip their body in the water as it causes deep rashes on the skin. But now they are used to this, so they go in sometimes, when they absolutely need to. However, if you [referring to the researcher] suddenly dip your feet into the water, your skin will be badly affected.

A key stakeholder highlighted the occupational health hazards of landfill workers due to the lack of safety equipment, and likelihood of being infected with diseases such as jaundice and hepatitis B. The findings reflected that, overall, improper management of waste has adverse effects on those working in or living near the landfills.

## 4 Discussion

This study described the overall scenario of Dhaka’s two landfills using qualitative and spatial techniques. It explored the environmental and health effects of the Matuail and Aminbazar landfills’ waste management processes along with the leachate treatment and managerial practices of the landfills. The inclusion of such wider thematic areas differentiates it from other studies while adding to the existing growing literature on landfill waste treatment.

A multi-ring buffer zone from the landfill was deployed to determine its influence over the surrounding features. The proximity to different land cover features is considered a significant determinant of exposure. This is also supported by the qualitative findings indicating that the adjacent agricultural and water bodies, roads, and settlements were affected by landfill pollutants. According to a guideline (Central Pollution Control Board 2017) a landfill must be located 250–300 m from habitats. Subsequently, the water bodies and roads also must be 200 m from the landfill. Besides, Guiqin *et al*. (2009) and Sener *et al*. (2010) reported that a landfill should not be sited within 500 m of water bodies. However, water bodies and agricultural lands were found within 300 m of the observed landfills, which are extremely susceptible to pollution. Settlements and roads were found to be located within the 500 m buffer zone. Especially in the Matuail landfill, the settlements and roads were located at a significantly closer distance to the disposal site. The above-mentioned studies also highlighted the severe exposure of land features and settlements located in the immediate vicinity by landfill pollutants.

A lack of proper management practices was revealed at the Aminbazar landfill due to a lack of adequate land availability and number of employees. The absence of proper waste treatment, presence of putrid odour and mosquitoes, flies and other animals increase the risk of health ailments among the surrounding people. A previous study focusing on Rawalpindi, Pakistan, demonstrated the poor municipal SWM system due to a lack of proper equipment and funding. Adverse environmental effects were caused by exposing toxic gases to the atmosphere and percolating leachate-contaminated groundwater resources and posed serious public health threats (Ejaj *et al*. 2010). Alam & Ahmade (2013) reported the contamination of surrounding water and soil bodies by untreated leachate and uncontrolled MSW incineration contributing to urban air pollution, similar to the current study’s findings regarding waste incineration. This spontaneous incineration may be due to changes in moisture content, intrusion of air and high temperature load, usually initiated by solid materials with lower ignition points (Moqbel 2009). Another study suggested the incineration of waste and energy supply are dependent on each other (Eriksson & Finnveden 2017), reflecting the proposed incineration plant at Aminbazar is a good step forward for Dhaka. However, while incineration is a positive step towards effective waste management and decreases quantity of waste, it has significant adverse health impacts including a higher incidence of cancer and respiratory symptoms. It also contributes towards detrimental environmental effects, including increased carbon emissions, acidification, an increase of heavy metals and toxic ash disposal, thereby increasing human exposure to toxic pollutants (Sharma *et al*. 2013).

Insufficient budgetary allocation was found to be the main impediment towards conducting proper SWM practices. Similarly, another previous study demonstrated that lack of landfilling process, budget constraints, and improper choice of technology hinder appropriate municipal SWM in Bangladesh (Abedin & Jahiruddin 2015). More recent research results showed a strong linkage between the lack of equipment for waste management activities and management planning with environmental or health issues (Ferronato & Torretta 2019).

Regarding waste leachate treatment, the findings indicated treatment plants mitigated the degree of pollution by refining the leachate, but the percolation system and mechanism did not take into account protecting the nearby agricultural land or groundwater from pollution. Previous studies showed that uncontrolled and untreated leachate of a landfill site polluted the surrounding soil, surface water and groundwater (Azim *et al*. 2011; Bhuiya *et al*. 2002). However, Azim *et al*. (2011) found local people and fishermen using the surrounding low-land areas of the southern and eastern sites of the Matuail landfill for fish cultivation. Such a practice was unobservable during this study, which may be because excessive water pollution has prohibited fish cultivation.

Other similar studies revealed that land adjacent to landfill areas, usually used to cultivate crops by the local people, might accumulate hazardous substances from the decomposed waste and leachate from the dumping site (Haque *et al*. 2013; Hossain *et al*. 2018), confirming the findings. A prior study of the Matuail landfill showed that leachate overflow may contaminate nearby crops (Hossain *et al*. 2018), supporting this study’s findings. The risk of polluting the surrounding lowlands (used for agriculture and fisheries) is particularly high during the monsoon due to heavy rainfall causing a greater flow of drainage water/leachate (Azim *et al*. 2011; Haque *et al*. 2013; Hossain *et al*. 2018). This study also noted that crop cultivation near the Matuail landfill is less than stated in previous studies.

Odour pollution was observed due to uncovered waste. This poses a threat to air quality. It also stimulates the breeding of vectors such as mosquitoes and flies. Many people living beside the Aminbazar landfill were driven to relocate due to the terrible landfill odour. These findings are supported by a South Africa-focused study (Njoku *et al*. 2019) that revealed some dwellers near the landfill regularly kept the doors and windows of their houses closed to prevent the entry of mosquitoes and rodents. Danthurebandara *et al*. (2012) also found that due to the disturbance of the flies, odour, smoke and noise, people do not want to reside close to landfills.

Dhaka landfill workers are at risk of many injuries and health hazards due to the manual segregation of waste without using protective gear. This health risk is substantiated by Ang *et al*. (2013), who stated that the manual process of sorting wastes poses a high risk of becoming infected with diseases. Additionally, the landfill waste management practices have an adverse effect on the health of nearby dwellers, as reported by many FGD participants. A majority of the landfill workers and nearby people suffer from health ailments such as pneumonia, skin rashes, loss of appetite, headache and stomach problems. These findings are supported by Norsa’adha *et al*. (2020), Njoku *et al*. (2019) and Vrijheid (2000), who documented eye irritation, skin rashes, nasal irritation, headache, excessive tiredness, excessive day sleepiness, sore throat, diarrhoea and stomach ache; and diseases such as asthma, tuberculosis, pneumonia, typhoid fever, dengue fever, cholera, food poisoning, cancer, hepatitis A, epilepsy, hypertension and diabetes mellitus among residents living near the open dump sites.

## 5 Limitations

Although this study revealed many novel findings, it also has several limitations. Qualitative data were the primary source, supported in a limited capacity by spatial data. It was not possible to collect quantitative data primarily due to time and budget constraints. Available spatial data were collected from Survey of Bangladesh (SoB), but a lack of microlevel data led to the use of poor-resolution satellite images and Google maps to identify the natural and social features of particular areas. Challenges were also faced during primary data collection. It was difficult to ensure all FGD respondents were able to participate equally and express their opinions about the study topics. The absence of a permanent landfill official at Aminbazar also impeded comprehensive data collection. It was also not possible to scientifically ascertain the water, soil and air contaminations of the environment surrounding the landfill and their effects on the health morbidity of dwellers near the landfills.

## 6 The Way Forward

The findings reveal that the existing SWM practices in Dhaka city can potentially become better organised and effective. The existing waste management system requires modification to ensure environmental sustainability and equitable public health. To reduce the waste quantity at the landfill, better primary waste disposal practices are needed. It is imperative to increase awareness among the people engaged in waste activities about the adverse effects from the uncontrolled dumping of solid waste through the media: newspaper advertisements, billboards, brochures and leaflets. Enlightenment campaigns, clean-up campaigns and recycling campaigns are ways of creating awareness. Past studies have shown that the integrated use of all media can increase public participation in solid waste recycling (Omran *et al*. 2009).

Imposing penalties due to improper SWM practices can also be a suitable approach, as practiced by many local governments, including California, which imposes a maximum daily penalty of US$70,000 for improper hazardous waste disposal (Waste360 2018). This is also mentioned as a possible beneficial practice for low-income countries such as Bangladesh in a UN-Habitat report (Adom & Kwach 2012).

For landfills, it is necessary to prevent the leachate from leaking and mixing with the surrounding agricultural land and river, possibly by establishing a fixed protective barrier. Beside a fixed boundary, a proper basement and leachate suction are necessary to control the leachate’s flow. The organisational capacity of the landfills must also be improved, though sufficient technical and manpower support and ensuring enough budget allocation for proper SWM procedures. It is also necessary to improve the current aerobic leachate treatment process through low-tech, simple and innovative methods.

While it is necessary to increase the landfill land area, waste generation growth due to increasing urban population will exhaust the additional land in five to seven years. The waste treatment and recycling process must be waste reductive and adapted according to waste disposition. Alternative practices such as incineration and the creation of refuse-derived fuel (RDF) should be explored especially for their environmental and health impacts. Only the residue waste after composting and burgess conversion should be disposed at the landfill. Ganesh *et al*. (2013) suggested the establishment of incineration and RDF plants. They found RDF to be an effective way of handling municipal waste, which leads to the creation of a green environment. This practice can reduce the burden of using fossil fuels in Bangladesh as well. About 20% of waste disposed can be reduced in this way, extending the service life up to 20–25 years. To further reduce the waste, the landfill authorities should establish a formal recycling biogas plant. The employees of this plant must be under the city corporation, and middlemen should be removed from the system. Vulnerable people (pregnant, ill or aged) and children should not be allowed to work in dangerous recycling activities; employees may be hired based on their skill and number of dependants.

Landfill gas (LFG) has an unpleasant stench that can cause headaches and nausea for landfill workers and nearby residents. Methane (CH_4_), the main component of LFG, can be used as a natural gas easily. Therefore, the landfills must capture and convert the LFG into a renewable energy resource to ensure environmental sustainability. Environmentally friendly treatments, such as waste-to-fertiliser conversion, eco-friendly leachate treatment, tunnel composting, *etc*. should be introduced to reduce and remove the landfill odour. A special ecofriendly landfill in Denton, Texas, with ‘mining’ can be a good practice for Bangladesh. This landfill uses a machine within the long-established tip or rubbish pile to find metals, plastics and other materials to recycle and also to prevent soil contamination. This landfill also has a special organic waste treatment plant to collect the produced methane and use it for electricity generation for around 1600 households (Newton 2016). By addressing environmental concerns, steps should be taken to reduce the uncontrolled breeding of mosquitoes, flies and other insects, and birds and rodents.

Health risks should be reduced by providing adequate safety equipment such as masks, gloves and protective boots to landfill employees. However, Jerie (2016) found workers supplied with safety gear may not use it due to lack of comfort and awareness about safety issues. While regular training programmes are conducted to make the waste handlers aware about the importance of safety gear, through government, non-government and joint initiatives, enforcement of the rules remains the main challenge. Bureaucratic challenges also prevail, with the central government being responsible for developing necessary laws and regulations, but city corporation authorities responsible for implementing the action plans related to policies and laws. Therefore, the provided safety equipment should be comfortable and convenient with regular training programmes and monitoring activities to ensure the proper utilisation of safety gear.

## 7 Conclusions

Urban waste management strategies must take full account of the complex interactions that occur with a variety of urban systems. The policies and practices of urban waste management can have negative environmental and public health impacts. This study concludes that residents living near the two studied Dhaka landfill sites face higher environmental and health risks. Although these landfills have improved during the last few years, significant improvement is needed for the landfills to treat the waste in a sustainable and healthy manner. This evidence can assist Dhaka’s urban decision-makers and the public with understanding about the imperatives for improving health and sustainability.

## Data Availability

The qualitative data collected and analysed during this study are not publicly available for ethical reasons, but are available from the corresponding author on reasonable request. The spatial data are available through Google Maps and Open Street Maps. Some shapefiles were obtained with permission from the Survey of Bangladesh (SoB) and can be made available from authors upon reasonable request, with permission of the SoB.

## Practice Relevance

Waste management practices impact directly on environmental and public health. Dhaka’s existing waste management practices can become better organised to address these concerns. Source waste reduction must be implemented to ensure the reduction of disposed waste at landfills. Administrative capacity of landfills requires improvement to ensure proper waste management procedures. Increased awareness is also needed among the general population and waste handlers about the adverse effects of uncontrolled waste dumping, along with enforcement measures for improper waste practices. Application of a low-tech, simple and innovative method to improve the leachate treatment process is also needed. Environmentally friendly treatments should be considered in order to minimise landfill odour. Regular training programmes and monitoring activities are needed to ensure the proper utilisation of comfortable and convenient safety gear and reduce vector breeding.
